# Cigarette smoking during pregnancy and preeclampsia risk: a systematic review and meta-analysis of prospective studies

**DOI:** 10.18632/oncotarget.6190

**Published:** 2015-10-20

**Authors:** Jun Wei, Cai-Xia Liu, Ting-Ting Gong, Qi-Jun Wu, Lang Wu

**Affiliations:** ^1^ Department of Obstetrics and Gynecology, Shengjing Hospital of China Medical University, Shenyang, China; ^2^ Department of Clinical Epidemiology, Shengjing Hospital of China Medical University, Shenyang, China; ^3^ Center for Clinical and Translational Science, Mayo Clinic, Rochester, MN, USA

**Keywords:** cigarette smoking, meta-analysis, preeclampsia, risk factor

## Abstract

Although evidence from epidemiological studies evaluating the association between cigarette smoking during pregnancy and preeclampsia risk has been systematically reviewed, the findings have been out of date. To further clarify the relationship, we conducted this comprehensive meta-analysis of prospective studies. We searched PubMed and Web of Science up to August 2015 to identify prospective studies that evaluated the association between cigarette smoking during pregnancy and preeclampsia risk. Random-effects models were used to estimate summarized relative risk (RR) and 95% confidence intervals (CIs). Seventeen prospective studies involving 62,089 preeclampsia patients from a total of approximately 1.8 million subjects were included. Overall, there was a significant negative association between smoking during pregnancy and incidence of preeclampsia (*RR* = 0.67, 95% CI: 0.60–0.75), with significant heterogeneity (*I*^2^ = 91.7%). Such an inverse association was also detected in strata of subgroup analyses according to study location, study sample size, parity of populations, singleton pregnancy, and adjustment for potential confounders including maternal age, diabetes mellitus, chronic hypertension, body mass index, and gender of infant. In summary, this meta-analysis suggests that smoking during pregnancy is inversely associated with incidence of preeclampsia. Further large scale multi-center prospective studies are warranted to validate our findings.

## INTRODUCTION

Preeclampsia is characterized by either increased blood pressure and proteinuria or exaggerated inflammation of other organs [1, 2] and causes complications in 3–5% pregnancies globally [3], resulting in a large number of cases of morbidity and mortality for mothers and infants [3]. It is categorized into two subtypes according to the timing of onset of disease, early-onset and late-onset preeclampsia, which both represent serious complications [4, 5]. Although research has shown that genetic, environmental and vascular-mediated factors may jointly play roles in its pathogenesis [6, 7], the exact etiology remains unsatisfactorily understood.

Unlike the general knowledge regarding the harmful effects of smoking on health, maternal smoking during pregnancy has been demonstrated to protect potentially from the development of preeclampsia [8]. *In vitro* and *in vivo* studies have suggested that antiangiogenic factors may be involved in the pathogenesis of preeclampsia [9, 10]. Additionally, nicotine may not only reduce the plasma volume by influencing the production of prostaglandins [11, 12] but may also reduce the levels of oxidative stress by deregulating the antioxidant systems in the placenta [13, 14]. During the past several decades, many epidemiological studies have shown that smoking during pregnancy is inversely associated with incidence of preeclampsia [15–28]. Additionally, a dose-response relationship for the duration of smoking and risk of preeclampsia was detected in several studies as well [18, 22, 26]. On the other hand, such a significant association was not detected in several other studies [29–32]. England et al. [33] conducted a systematic review of this issue. However, that review did not quantitatively summarize the available evidence to provide an accurate estimation. Although there was a meta-analysis study which provided such a quantitative summary (summarized relative risk = 0.68, 95% confidence interval: 0.67–0.69 in cohort studies) [34], evidence was summarized only up to 1998, and information from many more updated original studies has not been incorporated [15–25, 29, 30]. An up-to-date comprehensive and quantitative meta-analysis summarizing all evidence is thus critical for more accurately assessing the aforementioned issue. Furthermore, considering that retrospective case-control studies have more severe biases due to their retrospective nature, we thus aimed to carry out the most comprehensive meta-analysis of prospective studies to evaluate systematically the relationship between smoking during pregnancy and risk of preeclampsia.

## RESULTS

### Literature search, study characteristics, and quality assessment

**Figure 1:**
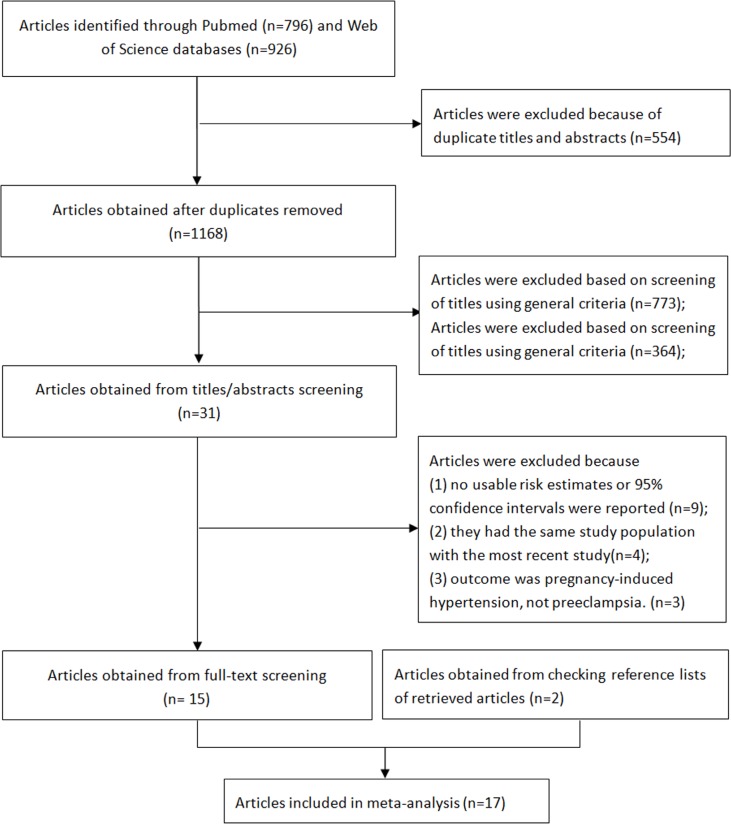
Flow-chart of study selection

The detailed steps of the literature search and article screening are shown in Figure 1. Briefly, a total of 1168 articles were retrieved after removing duplicates. After screening of titles and/or abstracts using general criteria, 1137 articles were excluded, leaving 31 articles for full text screening. Among them, sixteen articles were further excluded due to the following reasons: i) no usable risk estimates or 95% confidence intervals were reported; ii) study population duplication; and iii) the outcome was pregnancy-induced hypertension but not preeclampsia. Besides a total of 15 articles retained from the screening, we further identified two eligible articles from checking reference lists of retrieved articles [19, 26]. Overall, a total of 17 articles were included in the current meta-analysis [15–31].

The detailed characteristics of these included studies are shown in Supplementary Table S1. Overall, nine studies were conducted in Europe [16, 18–20, 22, 23, 25, 27, 31], six in the United States [15, 17, 21, 26, 28, 30], one in Canada [24] and another in Japan [29]. These studies enrolled 62,089 patients from a total of approximately 1.8 million subjects. Among these studies, nine reported the risk estimates as odds ratio (OR) [16–21, 23–25], five as relative risk (RR) [26–28, 30, 31], and one as hazard ratio (HR) [15]. The majority of included prospective studies adjusted for maternal age (*n* = 14). Less than half of the studies adjusted for diabetes mellitus (*n* = 5), chronic hypertension (*n* = 5), body mass index (*n* = 6) and gender of infant (*n* = 3).

The information of study quality is summarized in Table 1. Briefly, for representativeness of the exposed cohort, three studies [22, 23, 29] were not assigned a score because the included population came from a single hospital. For outcome of interest not present at the start of study, three studies [21, 22, 24] were not assigned a score because preeclampsia was presented at the beginning of the studies. For control for important factor or additional factor, nine studies [15–19, 26, 27, 30, 31] were assigned two scores in one column because they adjusted for more than two relevant factors. Therefore, the major issue of included studies might have been the adjustment for potential confounders in their primary analyses.

**Table 1:** Methodological quality of prospective studies included in the meta-analysis*

First author (reference), publication year	Representativeness of the exposed cohort	Selection of the unexposed cohort	Ascertainment of exposure	Outcome of interest not present at start of study	Control for important factor or additional factor^†^	Assessment of outcome	Adequacy of follow-up of cohorts^‡^
Lisonkova et al [15], 2013	*	*	*	*	* *	*	*
Perni et al [16], 2012	*	*	*	*	* *	*	*
Stone et al [17], 2007	*	*	*	*	* *	*	*
Hammoud et al [18], 2005	*	*	*	*	* *	*	*
Ioka et al [29], 2003	—	*	*	*	—	*	*
Basso et al [19], 2003	*	*	*	*	* *	*	*
England et al [30], 2002	*	*	*	*	* *	*	*
Newman et al [21], 2001	*	*	*	—	*	*	*
Mortensen et al [20], 2001	*	*	*	*	*	*	*
Xiong et al [24], 2000	*	*	*	—	*	*	*
Odegard et al [23], 2000	—	*	*	*	*	*	*
Martin et al [22], 2000	—	*	*	—	—	*	*
Lindqvist et al [25], 1999	*	*	*	*	*	*	*
Zhang et al [26], 1999	*	*	*	*	*	*	*
Knuist et al [31], 1998	*	*	*	*	*	*	*
Cnattingius et al [27], 1997	*	*	*	*	*	*	*
Coonrod et al [28], 1995	*	*	*	*	—	*	*

* A study could be awarded a maximum of one star for each item except for the item Control for important factor or additional factor. The definition/explanation of each column of the Newcastle-Ottawa Scale is available from (http://www.ohri.ca/programs/clinical_epidemiology/oxford.asp.).

† A maximum of 2 stars could be awarded for this item. Studies that controlled for maternal age received one star, whereas studies that controlled for other important confounders such as socioeconomic status/education, body mass index received an additional star.

‡ A cohort study with a follow-up rate > 70% was assigned one star.

### Smoking during pregnancy and preeclampsia

**Figure 2:**
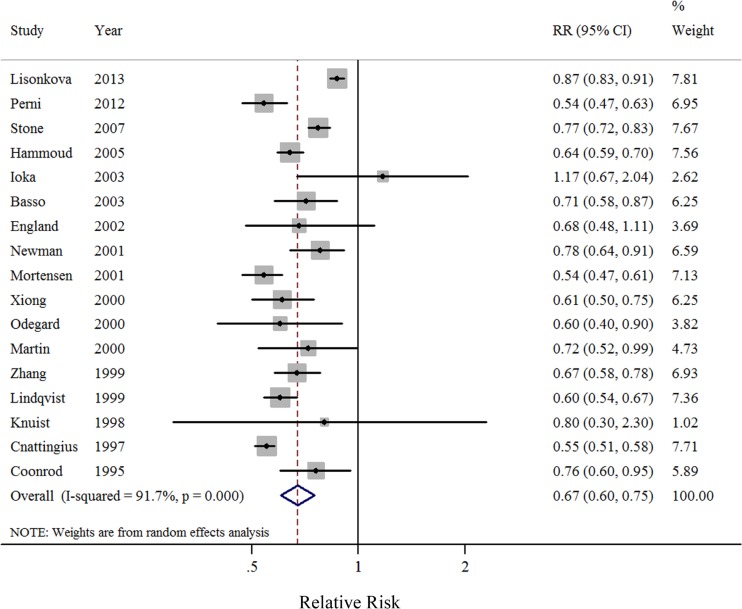
Forest plots (random effect model) of meta-analysis on the relationship between cigarette smoking during pregnancy and incidence of preeclampsia Squares indicate study-specific risk estimates (size of the square reflects the study-specific statistical weight); horizontal lines indicate 95% CIs; diamond indicates the summary relative risk with its 95% CI. RR: relative risk.

**Figure 3:**
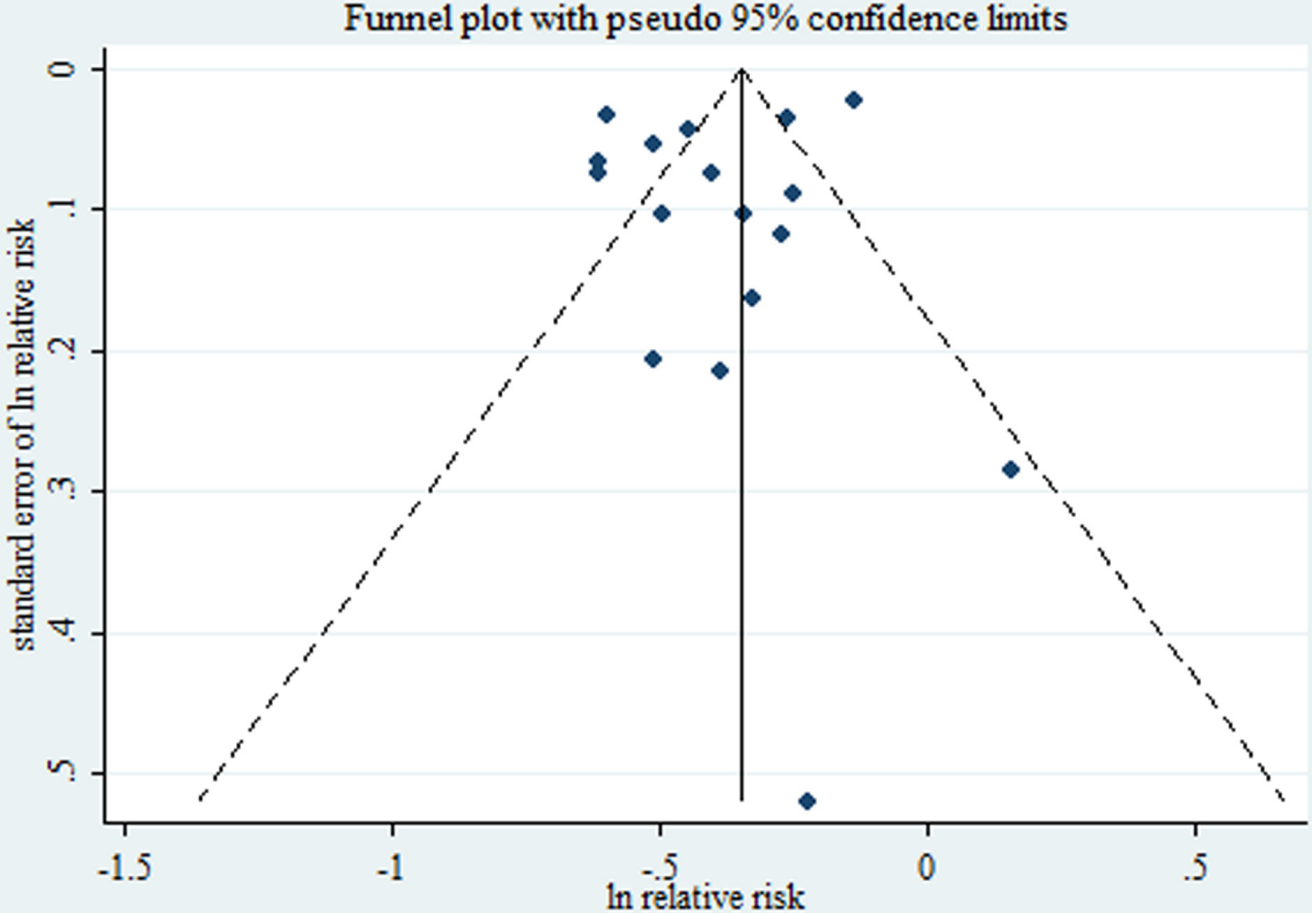
Funnel plot corresponding to the random-effects meta-analysis of the relationship between cigarette smoking during pregnancy and preeclampsia risk

After summarizing estimates from all available studies, there was a significant inverse association between smoking during pregnancy and incidence of preeclampsia (*RR* = 0.67, 95% CI: 0.60–0.75), with considerable heterogeneity (*I*^2^ = 91.7%, *P* < 0.001, Figure 2). There was no significant publication bias as indicated by Egger's test (*P* for bias = 0.358), Begg's test (*P* for bias = 0.232), and lack of asymmetry in the funnel plots when inspected visually (Figure 3).

### Subgroup and sensitivity analyses

Significant inverse associations were detected in almost all strata of subgroup analyses according to study location, study sample size, parity, type of pregnancy, and adjustment for potential confounders including maternal age, diabetes mellitus, chronic hypertension, body mass index, and gender of infant (Table 2). Notably, the results of meta-regression analyses demonstrated that study location and adjustment for chronic hypertension might have been the source of heterogeneity of the evaluated association. The observed association was stronger among studies in Europe compared with studies in the U.S. Similarly, the summarized estimates were significantly different in studies stratified by adjustment for chronic hypertension (*P* = 0.002).

**Table 2:** Summary risk estimates of the associations between smoking and preeclampsia risk

	No. of studies	Summarized RR	95% CI	*I*^2^ (%)	*P*^†^	*P*^‡^
**Overall**	17	0.67	0.60–0.75	91.7	< 0.01	
**Quality assessment**						0.90
High (≥ 9)	9	0.67	0.58–0.79	95.0	< 0.01	
Low (< 9)	8	0.66	0.58–0.75	64.9	0.01	
**Study location^§^**						< 0.01
North America	7	0.75	0.68–0.83	77.0	< 0.01	
Europe	9	0.59	0.55–0.64	50.2	0.04	
**Population size**						0.18
< 20000	8	0.72	0.66–0.79	0	0.57	
≥ 20000	9	0.64	0.55–0.74	95.7	< 0.01	
**Parity of study population**						0.17
Primiparas	5	0.70	0.63–0.76	0	0.56	
Multiparas	3	0.61	0.51–0.74	23.0	0.27	
**Singleton pregnancy**						0.29
Yes	12	0.66	0.58–0.75	93.7	< 0.01	
No	5	0.75	0.63–0.88	1.4	0.40	
**Adjustment for potential confounders**						
**Maternal age**						0.17
Yes	12	0.66	0.58–0.74	93.2	< 0.01	
No	3	0.79	0.65–0.96	13.9	0.31	
**SES/Education**						0.93
Yes	6	0.67	0.55–0.82	96.7	< 0.01	
No	11	0.66	0.60–0.72	53.3	0.02	
**Diabetes mellitus**						0.68
Yes	5	0.68	0.58–0.81	94.5	< 0.01	
No	12	0.65	0.59–0.72	68.0	< 0.01	
**Chronic hypertension**						< 0.01
Yes	5	0.80	0.73–0.88	64.2	0.03	
No	12	0.62	0.58–0.67	62.8	< 0.01	
**Body mass index**						0.51
Yes	6	0.63	0.58–0.69	25.3	0.24	
No	11	0.69	0.60–0.80	94.2	< 0.01	
**Infant's sex**						0.68
Yes	3	0.69	0.51–0.92	95.7	< 0.01	
No	14	0.66	0.60–0.73	82.1	< 0.01	

CI, confidence interval; N/A, not available; RR, relative risk; SES, socioeconomic status.

† *P* value for heterogeneity within each subgroup.

‡ *P* value for heterogeneity between subgroups with meta-regression analysis.

§ Excluding one study from Japan.

**Figure 4:**
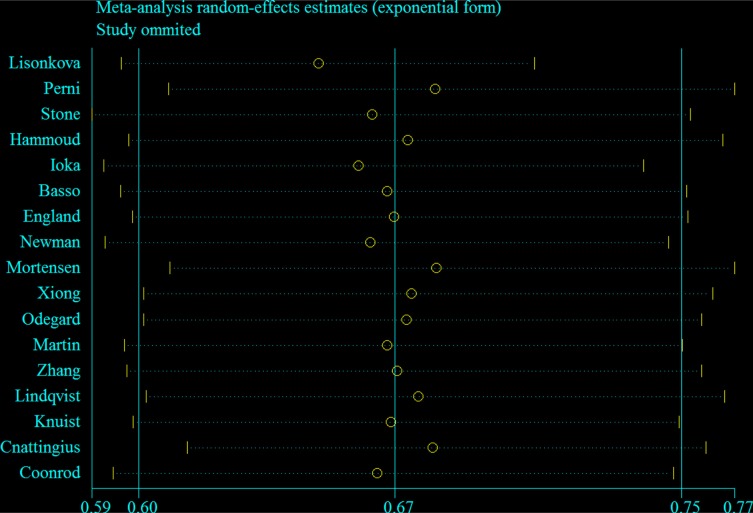
Sensitivity plot corresponding to the relationship between cigarette smoking during pregnancy and preeclampsia risk

According to the sensitivity analysis by omitting one study at a time, the summarized RRs ranged from 0.65 (95% CI: 0.60–0.71, *I*^2^ = 79.8%) after excluding the study by Lisonkova et al. [15] to 0.69 (95% CI: 0.61–0.77, *I*^2^ = 91.5%) after excluding the study by Mortensen et al. [20] (Figure 4). Additionally, when we excluded three studies [22, 28, 29] that provided the crude estimates without adjustment for any potential confounders, the summarized risk estimates remained similar (*RR* = 0.66, 95% CI: 0.58–0.74, *I*^2^ = 93.1%). Furthermore, considering that the incidence rate of preeclampsia is relatively high in some included studies [21, 22, 29], we used the method proposed by Zhang et al. [35] to convert the RRs into ORs for the summarized analysis, and the result indicated that our finding was robust (summarized *OR* = 0.67, 95% CI: 0.60–0.75, *I*^2^ = 92.1%).

## DISCUSSION

Considering the huge public health burden of preeclampsia as well as the lack of a summary of all available evidence on the relation between smoking during pregnancy and the incidence of this disease, we carried out this comprehensive systematic review and meta-analysis of prospective studies. Overall, the risk of preeclampsia decreased by 33% among women who ever smoked during pregnancy. Significant inverse associations were also observed in almost all strata of subgroup analyses.

Although the exact mechanism responsible for the relationship between smoking during pregnancy and incidence of preeclampsia is not completely understood, several potential biological mechanisms have been put forth, partly explaining this inverse association. Previous studies found that antiangiogenic factors such as soluble fms-like tyrosine kinase 1 (sFlt1; also known as soluble vascular endothelial growth factor receptor-1) and soluble endoglin (sEng) levels may play an important role in the pathogenesis of preeclampsia [9, 10]. In experimental studies, carbon monoxide, one of the main toxic chemicals of smoking, lowers sFlt1 and soluble endoglin production in endothelial cells and placental cultures through the heme oxygenase-1/carbon monoxide pathway [36]. Several studies have also suggested that non-smokers have a higher circulating level of sFlt1 than do cigarette smokers during pregnancy [10, 37]. Nicotine may reduce the plasma volume by influencing the production of prostaglandins, which are known to be vasoconstrictors [11, 12]. Furthermore, since antioxidant systems in the placenta are found to be deregulated among smoking women, the reduced levels of oxidative stress may result in a reduction in preeclampsia [13, 14]. On the other hand, there is a possibility that smoking can lead to preterm delivery thus decreasing the incidence of preeclampsia [17, 38, 39]. These understandings are well aligned with our finding that smoking during pregnancy can be inversely associated with preeclampsia risk.

The results of the meta-regression analysis demonstrated that study location might have been a source of heterogeneity (*P* = 0.025). Although significant inverse associations for ever exposure to cigarette smoking during pregnancy with preeclampsia risk were observed in the studies from the U.S. and Europe, the effect estimate based on studies carried out in the U.S. was slightly weaker than that in Europe. This pattern could have been partly attributed to a different prevalence of cigarette smoking during pregnancy among different populations. The average cigarette smoking rates in these included studies were 21.7 and 25.5% for the U.S. and Europe, respectively. However, there is little discrepancy in the mean incidence rates of preeclampsia between these two locations (5.4% for U.S. *versus* 5.1% for Europe).

Our study has several strengths. First, to the best of our knowledge, this study provided evidence of the relationship between smoking during pregnancy and preeclampsia risk on the basis of the most updated cohort studies, which was the main difference from the previous meta-analysis. Second, our meta-analysis included 17 cohort studies involving 62,089 patients from a total of approximately 1.8 million participants, which provided sufficient power to detect modest associations. Third, because of the prospective design of all included studies, the influence of several biases including recall bias and selection bias could be minimized which resulted in the high quality of these included studies. Furthermore, numerous subgroup analyses according to study characteristics and adjustment for potential confounders suggested that our findings were robust.

**Figure 5:**
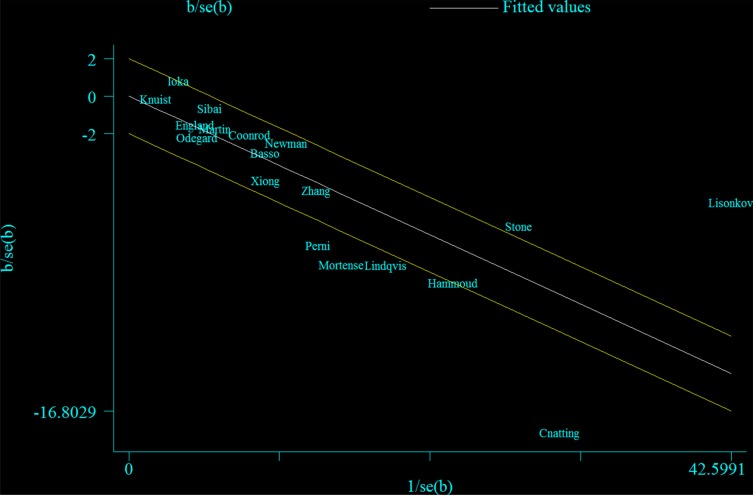
Galbraith plot corresponding to the random-effects meta-analysis of the relationship between cigarette smoking during pregnancy and preeclampsia risk

Several potential limitations must be acknowledged for our findings. First, we could not fully rule out the possibility of residual confounding because of the nature of observational studies. Although 14 of the 17 included studies provided adjusted estimates considering potential confounders, only the variable maternal age was regularly adjusted in the majority of included studies (*n* = 14). Among the 14 studies, nine adjusted for more than two important factors in their primary analyses. Even though the significant inverse associations persisted in all strata of subgroup analyses according to adjustment for potential confounders, the meta-regression analyses demonstrated that whether there was adjustment for chronic hypertension might have been the source of heterogeneity, though we could not rule out the possibility that this result was a chance finding because only 5 studies adjusted for this potential confounder. Furthermore, Perni et al. [16] indicated that maternal mood, anxiety disorders as well as partner change may confound the association between smoking during pregnancy and preeclampsia risk. However, only one included study [18] adjusted for part of these factors in the primary analyses. Since we did not have access to the primary data of these included studies, further pooled analysis is warranted to fully adjust for the potential confounders or report analyses stratified by these possible risk factors to rule out potential residual confounding. Second, significant heterogeneity could be a concern when interpreting the findings of this study. Although numerous subgroup and sensitivity analyses were carried out, heterogeneity still existed in our study. We performed a Galbraith plot to visualize these studies that could have generated the heterogeneity (Figure 5). After excluding seven studies [15–18, 20, 25, 27] from the scale of this plot, significant association persisted and there was no more heterogeneity (*RR* = 0.70, 95% CI: 0.65–0.76, *I*^2^ = 0%). Third, self-reported smoking during pregnancy may result in misclassification. This kind of misclassification may also widely exist in relevant studies, since females tend to change behaviors during early stage of pregnancy while the change may not be accurately captured in those studies [16, 24, 27, 29, 30]. England et al. [40] suggested that self-reported smoking habits of pregnant women may confer an exposure misclassification as high as 21.6% [16]. The study reported by George et al. [41] indicated that self-reported smoking lacked validity among women who stated that they recently had stopped smoking. Furthermore, only three included studies [23, 25, 26] carried out dose-response analysis, which restricted the possibility to evaluate the hypothesis that heavy smokers might have decreased risk of preeclampsia. Additionally, whether the aforementioned associations could be observed among past smokers needs further investigation.

In summary, the results of this most updated meta-analysis of prospective studies add to the weight of evidence suggesting that cigarette smoking during pregnancy is inversely associated with preeclampsia. Further prospective studies are warranted to adjust fully for potential confounders and to provide more detailed results stratified by smoking status and subtypes and severity of preeclampsia. Moreover, since the effective treatments of this disease are still lacking, more *in vivo* or *in vitro* studies are needed to understand the exact biological mechanisms of this aforementioned association which may contribute to identifying the appropriate therapeutic strategies.

## MATERIALS AND METHODS

### Search strategy

A comprehensive literature search of PubMed (MEDLINE) and Web of Science databases was conducted from each database's inception to August 2015 for relevant studies. We used the following search keywords: (smoking OR tobacco OR cigarette) AND preeclampsia AND pregnancy. We also screened references of relevant review articles and included studies to identify other potential studies [42–45]. This meta-analysis was planned, conducted, and reported in adherence to the Meta-analysis Of Observational Studies in Epidemiology (MOOSE) guidelines [46].

### Study selection and exclusion

Published studies were eligible if they met the following criteria: (i) they were prospective studies; (ii) they clearly defined preeclampsia as gestational hypertension and proteinuria; (iii) they evaluated the association between smoking during pregnancy and preeclampsia risk; and (iv) they presented relative risk (RR), odds ratio (OR), or hazard ratio (HR) estimates with 95% confidence intervals (CIs) or necessary data for determination. There was no restriction for sample size and follow-up duration. If several publications involved overlapping individuals, we included the study with the most patients.

Published studies were excluded for the following reasons: (i) they were non-epidemiological studies, retrospective studies, reviews without original data, ecological studies, editorials, or case reports; (ii) they reported risk estimates that could not be summarized (such as risk estimates without 95% CIs); and (iii) they reported the outcome as pregnancy-induced hypertension instead of preeclampsia.

### Data abstraction and quality assessment

A pair of investigators (JW and Q-JW) independently carried out the abstract screening, full-text screening, and data extraction. Disagreements were resolved by discussion. Data extracted from each study included: last name of the first author, publication year, study location, study period, characteristics of study population (sample size, parity information, and singleton or twin pregnancy categories), and effect sizes of the associations (including adjusted confounders information if applicable). If there were multiple estimates for the association, we used the estimate adjusted for the most appropriate confounding variables, like in previous studies [47, 48]. In situations when only unadjusted estimates were given, we used the unadjusted estimates.

To assess the methodological quality of all included studies, the Newcastle-Ottawa Scale (NOS) [49–53] was used in this meta-analysis. As we mentioned in previous studies [42, 48], since quality scoring may not only submerge important information by combining disparate study features into a single score but may introduce somewhat arbitrary subjective elements into the analysis [54–56], we evaluated these included studies on the basis of NOS instead of scoring them and categorizing them into high or low quality according to the scores.

### Statistical analysis

For studies that presented risk estimates separately by the subtype of preeclampsia [15], severity of preeclampsia [27], datasets [25], and status of parity [19], the fixed-effects model [57] was used to summarize the estimates to a combined estimate before incorporating it into the overall summarizing analysis. For studies that did not provide the exact risk estimates for ever *versus* never exposure to smoking during pregnancy [25, 26], we used the effective-count method proposed by Hamling et al. [58] to recalculate the risk estimates. We used *I*^2^ to evaluate the heterogeneity across studies, in which *I*^2^ > 50% suggests high heterogeneity and *I*^2^ ≤ 50% suggests low heterogeneity [59]. We summarized log-transformed RR using the random-effects model [60]. To investigate possible sources of heterogeneity of main findings, subgroup analyses were conducted by study location (U.S., Europe, and others), study quality (high *versus* low), mean sample size of the prospective study (< 20000 *versus* ≥ 20000), parity of study population (primiparas *versus* multiparas), singleton pregnancy (yes *versus* no), and adjustment for potential confounders including maternal age, socioeconomic status/education, diabetes mellitus, chronic hypertension, body mass index, and gender of infant. Heterogeneity between subgroups was evaluated by meta-regression [50, 51, 61].

Small study bias such as publication bias was evaluated with Egger's test [62] and Begg's test [63]. A *P*-value of 0.05 was used to determine whether significant publication bias existed. All statistical analyses were performed with Stata (version 12; StataCorp, College Station, TX).

## SUPPLEMENTARY MATERIALS TABLE


