# Supplementary material for: Cigarette smoking during pregnancy and preeclampsia risk: a systematic review and meta-analysis of prospective studies
Source: Oncotarget. 2015 Oct 20;6(41):43667–78. doi: 10.18632/oncotarget.6190 (PMC4791258; doi:10.18632/oncotarget.6190)
Supplement: Supplementary file 1 [file oncotarget-06-43667-s001.pdf]

Cigarette smoking during pregnancy and preeclampsia risk: a systematic review and meta-analysis of prospective studies

Supplementary Materials

Table S1: Characteristics of prospective studies included in the meta-analysis

First Author, (Reference), Year, Location	Study Period	No. of Case/ Cohort	Population characteristics, Incidence of PE	Risk Estimates (Ever *vs.* Never) (95% CI)	Adjusted factors
Lisonkova et al [15], 2013, USA	2003–2008	14201/456,668	Singleton delivery women, 3.1%	Early onset PE: 0.87 (0.82–0.93)<br>Late onset PE:0.87 (0.82–0.93)<br>Hazard Ratio	Age, race, education, marital status, number of prior live births, diabetes mellitus, chronic hypertension, infertility treatment, infant sex, congenital anomalies
Perni et al [16], 2012, Sweden	1992–2006	18891/371,627	Singleton delivery women, 5.1%	PE: 0.54 (0.47–0.63)<br>Odds Ratio	Age, country of origin, interpregnancy interval, BMI in second pregnancy, BMI change from first to second pregnancy, height, maternal education level, pregestational diabetes, gestational diabetes, and history of stillbirth
Stone et al [17], 2007, USA	2000–2001	7002/129,674	Singleton delivery women, 5.4%	PE: 0.78 (0.73–0.83)<br>Odds Ratio	Maternal age, maternal race, parity, medicaid coverage, Kotelchuck Index, chronic hypertension, diabetes mellitus, and renal disease
Hammoud et al [18], 2005, Germany	1991–1997	3736/170,254	Singleton delivery women, 2.2%	PE: 0.64 (0.59–0.70)<br>Odds Ratio	Age, BMI, social and psychologic stress, previous termination of pregnancy, preterm labor, preexisting diabetes mellitus, gestational diabetes mellitus, parity, and infertility treatment
Ioka et al [29],[†] 2003, Japan	1997–1998	85/493	Pregnant women, 17.2%	PE: 1.17 (0.67–2.04)<br>Odds Ratio	N/A

Basso et al [19], 2003, Denmark	1998–2001	784/44,732	Singleton delivery women, 1.8%	Primiparas with reported hypertension PE: 0.76 (0.44–1.18)<br>Primiparas without reported hypertension PE: 0.70 (0.53–0.92)<br>Multiparas with reported hypertension PE: 0.75 (0.44–1.28)<br>Multiparas without reported hypertension PE: 0.65 (0.38–1.12)<br>Odds Ratio	Age at delivery, BMI, irregular cycle, times to pregnancy
England et al [30], 2002, USA	1992–1995	326/4589	Primigravidas pregnant women, 7.1%	PE: 0.7 (0.5–1.1)<br>Relative Risk	Maternal age, race, type of health insurance, study center, and BMI at the time of study enrollment
Newman et al [21], 2001, USA	1980–1989	2356/18,370	Primigravidas singletons delivery women, 12.8%	PE: 0.78 (0.64–0.91)<br>Odds Ratio	Age, race and chronic hypertension
Mortensen et al [20], 2001, Denmark	1991–1998	1718/46,313	Singleton delivery women, 3.7%	PE: 0.54 (0.47–0.61)<br>Multiparas PE: 0.55 (0.46–0.66)<br>Odds Ratio	Maternal age, former stillbirth, mother living alone, parity, mother on insulin treatment before pregnancy, mother receiving cardiovascular medicine before pregnancy, and mother receiving antibiotics during first and/or second trimester
Xiong et al [24], 2000, Canada	1995–1997	645/58,216	Singleton delivery women, 1.2%	PE: 0.61 (0.50–0.75)<br>Primiparas PE: 0.63 (0.50–0.80)<br>Multiparas PE: 0.72 (0.51–1.02)<br>Odds Ratio	Maternal age, parity maternal weight, maternal alcohol consumption, drug dependence, prior intrauterine growth restriction and fetal anomaly, bleeding after 20 weeks, gestational diabetes mellitus, severe anemia and infant's sex
Odegard et al [23], 2000, Norway	1993–1995	323/12,804	Pregnant women, 2.5%	PE: 0.6 (0.4–0.9)<br>Mild PE: 0.5 (0.3–0.9)<br>Moderate PE: 0.7 (0.4–1.2)<br>Severe PE: 0.5 (0.3–0.9)<br>Early onset PE: 0.9 (0.4–2.2)<br>Late onset PE: 0.5 (0.4–0.8)<br>Primiparas PE: 0.6 (0.4–1.0)<br>Odds Ratio	Maternal age, maternal weight, blood pressure, nulliparous, preeclampsia in a previous pregnancy for parous women, multiple birth pregnancy

Martin et al [22],† 2000, United Kingdom	1969–1997	205/1511	Twin delivery women, 13.6%	PE: 0.72 (0.52–0.99) Odds Ratio	N/A
Lindqvist et al [25],‡ 1999, Sweden	1990–1994	3146/127,721	All singleton delivery women, 2.5%	PE: 0.6 (0.54–0.67) Odds Ratio	Maternal age, parity, fetal gender
Zhang et al [26],‡ 1999, USA	1959–1965	632/9651	Primigravidas singletons delivery women, 6.5%	PE: 0.67 (0.58–0.78) Relative Risk	Age, race, socioeconomic status, and pre-pregnancy BMI
Knuist et al [31], 1998, Dutch	1992–1994	34/2413	Primigravidas singletons delivery women, 1.4%	PE: 0.8 (0.3–2.3) Relative Risk	Age, BMI, blood pressure, abortion history
Cnattingius et al [27], 1997, Sweden	1987–1993	7450/317,652	Primigravidas singletons delivery women, 2.3%	Mild PE: 0.6 (0.5–0.6) Severe PE: 0.5 (0.5–0.6) Relative Risk	Maternal age, education, country of birth, and family situation
Coonrod et al [28], 1995, USA	1984–1988	555/11,694	All singleton and twin delivery women, 4.7%	Singleton PE: 0.7 (0.5–1.0) Twin PE: 0.8 (0.6–1.1) Relative Risk	N/A

BMI, body mass index; CI, confidence interval; N/A, not available; PE, preeclampsia.

†Risk estimates and 95% CI were calculated from published data with EpiCalc 2000 software (version 1.02; Brixton Health).

‡Risk estimates and 95% CI were converted by the method proposed by Harmling et al [58].
